# Human embryo models: unveiling sophisticated self-organization of stem cells during post-implantation stages

**DOI:** 10.1038/s41392-023-01677-0

**Published:** 2023-11-06

**Authors:** Manqiang Lin, Michael Sigal

**Affiliations:** 1 Department of Hepatology and Gastroenterology, Charité Universitätsmedizin Berlin, 13353 Berlin, Germany; 2 Berlin Institute for Medical Systems Biology (BIMSB), Max Delbrück Center for Molecular Medicine, 10115 Berlin, Germany

**Keywords:** Reprogramming, Pluripotent stem cells

In three recent articles published in *Nature* and *Cell*, Weatherbee et al.^1^, Pedroza et al.^2^ and Liu et al.^3^ have demonstrated how human pluripotent stem cells can be coaxed to self-organize into compartmentalized structures that mirror post-implantation embryos. Building on the successful establishment of stem cell-derived mouse embryo models ex utero, these models now shed light on human embryo development during the period between implantation and gastrulation, which has so far been challenging to investigate.

**Fig. 1:**
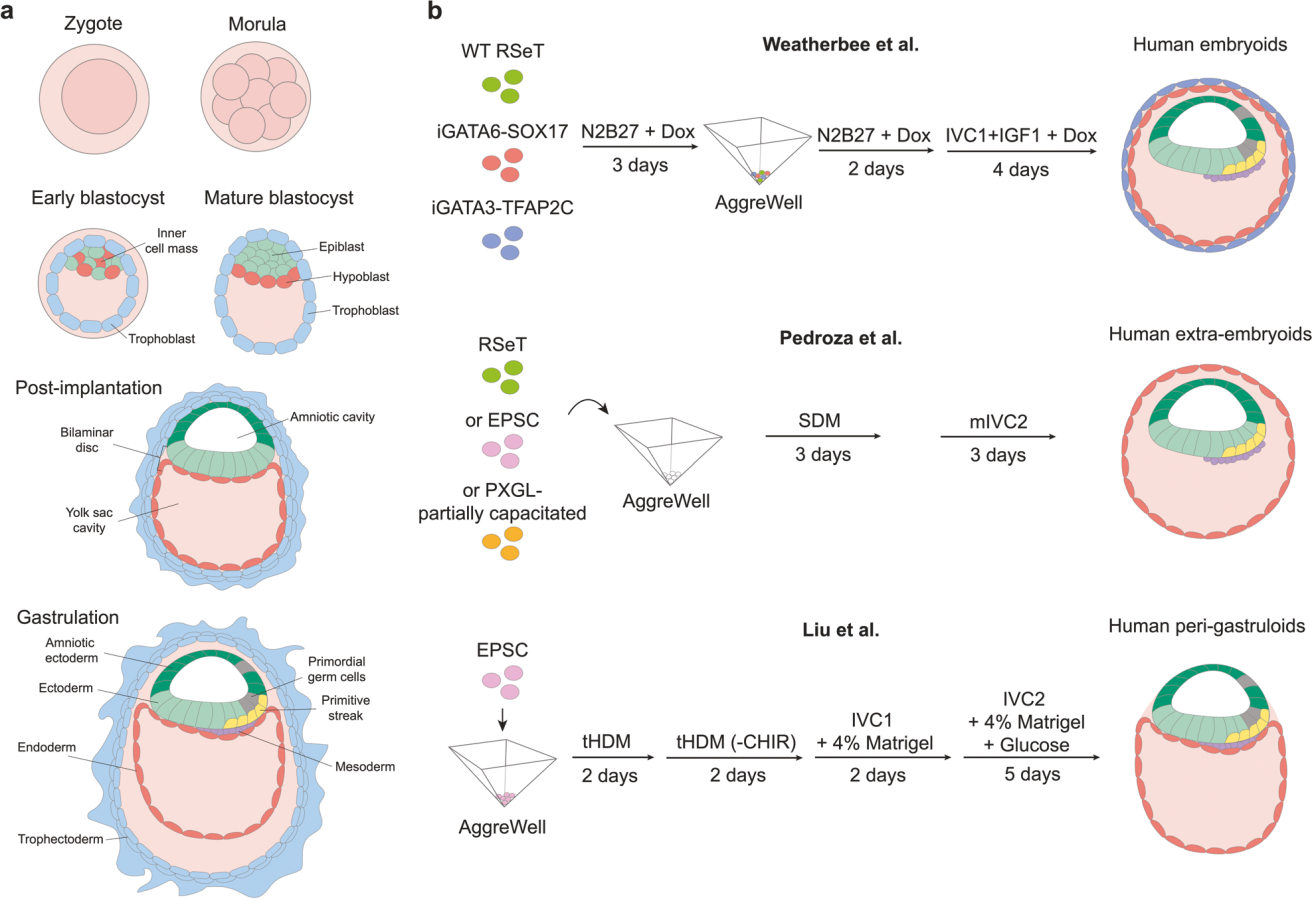
Peri-gastrulation development of human embryo and human stem cell-derived post-implantation models (the figure was created using Adobe Illustrator 26.3.1). **a** Schematic showing human developmental stages from pre-implantation to post-implantation including gastrulation. **b** Schematic for the establishment of human post-implantation models. Weatherbee et al. induced overexpression of hypoblast-specific genes *GATA6* and *SOX17* or trophoblast genes *GATA3* and *TFAP2C* in intermediate peri-implantation-like (RSeT) cells using doxycycline (Dox) for three days in N2B27 medium. Wild-type (WT) RSeT cells, iGATA6-SOX17 cells, and iGATA3-TFAP2C cells were then aggregated in AggreWell plates, cultured with N2B27 medium and continued Dox treatment. After 2 days, cell aggregates were transferred to in vitro culture medium 1 (IVC1) supplemented with IGF1 and Dox, and cultured for 4 more days. Pedroza et al. cultured RSeT cells, pre-implantation-like (PXGL-partially capacitated) cells, or extended pluripotent stem cells (EPSCs) in AggreWell plates with spontaneous differentiation medium (SDM) for 3 days. Cells were transferred to modified in vitro culture medium 2 (mIVC2) and cultured for 3 more days. Liu et al. cultured EPSCs in AggreWell plates with ‘titrated” hypoblast differentiation medium (tHDM) for 2 days and removed CHIR99021 (CHIR) for the following 2 days. The cells were then transferred to IVC1 medium with 4% Matrigel for 2 days, followed by IVC2 medium supplemented with 4% Matrigel and extra glucose for around 5 days

Following the first few cell divisions after fertilization, the resulting blastomeres become fate-restricted and form a blastocyst, consisting of the inner cell mass, which gives rise to the embryo, and the surrounding trophectoderm, an extraembryonic tissue that gives rise to the placenta (Fig. 1a). The inner cell mass then differentiates further into the epiblast – giving rise to the fetal tissues – and the hypoblast – a second extraembryonic tissue that gives rise to the yolk sac (Fig. 1a). Although they are derived from the inner cell mass of the blastocyst, previous work has shown that mouse embryonic stem cells (ESCs) can be used to generate in vitro models that give rise to both embryonic and all extraembryonic tissues and resemble gastrulation and early organogenesis. Indeed, naïve human ESCs (hESCs), which are hypomethylated and thus not primed for lineage commitment, can be used to generate blastocyst-like structures containing embryonic/extraembryonic tissues and have very recently been shown to progress to post-implantation stages when embedded in extracellular matrix.^4^

Instead of starting off with naïve hESCs, however, the three new models for post-implantation embryos utilized hESCs in intermediate pluripotency states and bypassed the blastocyst stage. The main problem posed by this approach consists of the different signaling factors required to induce the different lineages. Weatherbee et al.^1^ approached this challenge by first inducing extraembryonic lineages separately through transient overexpression of transgenes (Fig. 1b). After screening different transcription factors and medium conditions, they used GATA6-SOX17 and GATA3-TFAP2C to program hESCs into hypoblast-like and trophoblast-like states, respectively. The cells were cultured in RSeT medium, which maintains them in a peri-implantation-like state. After inducing expression of the selected transgenes for three days, the authors aggregated them with wild-type hESCs. The resulting “embryoids” self-organized into structures featuring an epiblast domain containing a central lumen, an intermediate hypoblast domain, and an outer layer of trophoblast-like cells. Embryoids formed with an efficiency of 23% and could be cultured for 6–8 days. Single-cell RNA-sequencing (scRNA-seq) and transposase-accessible chromatin sequencing revealed significant parallels between native embryos and the putative amnion, hypoblast, and extraembryonic mesenchyme cells. Although a distinct trophoblast-like population was not identified, the GATA3-TFAP2C cells secreted BMP, a key driver of primordial germ cell and amnion differentiation. Intriguingly, SOX17 overexpression, utilized to drive a hypoblast-like identity, obstructed the formation of hypoblast-derived anterior visceral endoderm, a critical signaling center for anteroposterior patterning. These findings demonstrate the value of such modular embryoid models for revealing the genes required to determine specific tissues. However, the gene modulation used may also override some of the complex signaling cross-talk between cells that drive self-organization, leading to deficiencies in lineage differentiation.

This is highlighted by the superior patterning observed in the model by Pedroza et al.^2^, who used different medium conditions (RSeT, EP, or partially capacitated PXGL) to induce intermediate pluripotency states (Fig. 1b). The resulting cells were then cultured as aggregates in minimum growth factor conditions to allow for spontaneous differentiation. Within 48 h, they underwent lineage segregation resembling epiblast-hypoblast patterning. On day 4, they were switched to medium optimized for post-implantation embryos, which led to efficient (~79%) formation of “human extra-embryoids” (hEEs), encompassing an inner epiblast-like and an outer hypoblast-like compartment, even in the absence of trophoblast-like cells. scRNA-seq revealed a sequential progression within the embryonic compartment, including the development of amnion-like and primitive streak-like cellular states. By day 6, an embryonic mesoderm-like state emerged, alongside cells resembling later, differentiated embryonic states, although definitive endoderm was not observed. Of note, they identified a transient state within the hypoblast lineage marked by elevated expression of antagonists for BMP, NODAL and FGF signaling, key indicators of anterior visceral endoderm. The model effectively orchestrated temporally organized expression domains resembling the amnion, which is BMP-dependent and reliant on the surrounding hypoblast. Importantly, hypoblast organization in hEEs hinged on NODAL and FGF activity. Although global epigenomic reprogramming was not observed, the system efficiently recapitulated key hallmarks of human peri-gastrulation, and shed light on the signaling crosstalk driving co-development of multiple early lineages.

In the third study, Liu et al.^3^ optimized culture medium conditions for development of a comparatively straightforward method to generate “peri-gastruloids” from extended pluripotent stem cells (EPSCs), which show potency for both embryonic and extraembryonic tissues (Fig. 1b). The model worked with EPSCs generated from several different human ES and iPS cell lines via a standard chemical cocktail. The authors’ success hinged on titrating a low dose of MEK inhibitor to optimize the FGF signaling activity of hypoblast differentiation medium (tHDM), enabling EPSCs to differentiate into both hypoblast and epiblast cells. Cells were then allowed to aggregate and sort into the two lineages. After 4 days, lumen formation was evident in both compartments and aggregates were transferred to morphogen-free IVC medium supplemented with 4% Matrigel. This was sufficient to support further development, directed solely by signaling factors produced within the peri-gastruloids themselves. On day 6, they exhibited well-defined structures, primordial germ cell specification and onset of gastrulation marked by expression of Brachyury. After 8 days, peri-gastruloids replicated symmetry breaking and anterior-posterior axis formation, offering insights into trilaminar disc and primitive streak formation along with the process of gastrulation in humans. Excitingly, in day 10–12 peri-gastruloids, morphology, the expression of marker genes and axis formation suggested the onset of neurulation and organogenesis with remarkable efficiency (~70%), going beyond the stages supported by the other two models. scRNA-seq analyses confirmed the presence of important cell types found in primate peri-gastrulation embryos, including epiblast, primitive streak, various types of ectoderm/mesoderm/endoderm, blood/vascular progenitors, as well as allantois and amniotic cell types. Although trophoblasts are clearly not essential for initial patterning and tissue maintenance, their lack may nonetheless result in some morphological abnormalities compared to native embryos. While this highlight was under review, Oldak et al.^5^ reported self-assembly of an embryo model grown in shaking culture that includes trophoblast-like cells derived from non-transgenic naïve hESCs. This did indeed support not only authentic morphological organization and orchestrated temporal progression but also a nearly full complement of developmental lineages, including most extra-embryonic compartments, albeit with a relatively low efficiency. Nonetheless, incomplete models that cannot develop into viable embryos may be better able to assuage ethical concerns around this kind of research while the regulatory frameworks catch up with these new developments.

Collectively, these sophisticated methodologies for establishing models of human post-implantation embryos have already illuminated the interplay between embryonic and extraembryonic compartments and now allow in-depth study of human development to advance reproductive and regenerative medicine. Notably, they share several technical and mechanistic concepts with sophisticated co-culture models based on adult stem cells that allow investigations into intercellular communication and self-organization in mature tissues. Much may be gained from synergizing the different strengths of these fields.
